# Gut microbiota-derived propionate mediates the neuroprotective effect of osteocalcin in a mouse model of Parkinson’s disease

**DOI:** 10.1186/s40168-020-00988-6

**Published:** 2021-01-31

**Authors:** Yan-fang Hou, Chang Shan, Si-yue Zhuang, Qian-qian Zhuang, Arijit Ghosh, Ke-cheng Zhu, Xiao-ke Kong, Shu-min Wang, Yan-ling Gong, Yu-ying Yang, Bei Tao, Li-hao Sun, Hong-Yan Zhao, Xing-zhi Guo, Wei-qing Wang, Guang Ning, Yan-yun Gu, Sheng-tian Li, Jian-min Liu

**Affiliations:** 1 Department of Endocrine and Metabolic Diseases, Ruijin Hospital, Shanghai Jiao Tong University School of Medicine, Shanghai Institute of Endocrine and Metabolic Diseases, Shanghai Clinical Center for Endocrine and Metabolic Diseases, Shanghai, 200025 China; 2 Bio-X Institutes, Key Laboratory for the Genetics of Developmental and Neuropsychiatric Disorders (Ministry of Education), Shanghai Key Laboratory of Psychotic Disorders, and Brain Science and Technology Research Center, Shanghai Jiao Tong University, Shanghai, 200240 China

**Keywords:** Osteocalcin, Parkinson’s disease, Gut microbiota, Propionate

## Abstract

**Background:**

Parkinson’s disease (PD) is a neurodegenerative disorder with no absolute cure. The evidence of the involvement of gut microbiota in PD pathogenesis suggests the need to identify certain molecule(s) derived from the gut microbiota, which has the potential to manage PD. Osteocalcin (OCN), an osteoblast-secreted protein, has been shown to modulate brain function. Thus, it is of interest to investigate whether OCN could exert protective effect on PD and, if yes, whether the underlying mechanism lies in the subsequent changes in gut microbiota.

**Results:**

The intraperitoneal injection of OCN can effectively ameliorate the motor deficits and dopaminergic neuronal loss in a 6-hydroxydopamine-induced PD mouse model. The further antibiotics treatment and fecal microbiota transplantation experiments confirmed that the gut microbiota was required for OCN-induced protection in PD mice. OCN elevated *Bacteroidetes* and depleted *Firmicutes* phyla in the gut microbiota of PD mice with elevated potential of microbial propionate production and was confirmed by fecal propionate levels. Two months of orally administered propionate successfully rescued motor deficits and dopaminergic neuronal loss in PD mice. Furthermore, AR420626, the agonist of FFAR3, which is the receptor of propionate, mimicked the neuroprotective effects of propionate and the ablation of enteric neurons blocked the prevention of dopaminergic neuronal loss by propionate in PD mice.

**Conclusions:**

Together, our results demonstrate that OCN ameliorates motor deficits and dopaminergic neuronal loss in PD mice, modulating gut microbiome and increasing propionate level might be an underlying mechanism responsible for the neuroprotective effects of OCN on PD, and the FFAR3, expressed in enteric nervous system, might be the main action site of propionate.

Video abstract

**Supplementary Information:**

The online version contains supplementary material available at 10.1186/s40168-020-00988-6.

## Introduction

Parkinson’s disease (PD) is the second most common neurodegenerative disorder and is clinically dominated by motor symptoms, including rigidity, resting tremor, and bradykinesia [1, 2] that result from the loss of dopaminergic neurons in the substantia nigra (SN), which is the cardinal pathological feature of PD [3]. Clinically, these motor symptoms respond well to dopaminergic replacement therapy [4]. However, this kind of treatment cannot reverse the progressive dopaminergic neurodegeneration in PD [5].

In recent years, the connection between gut microbiota and central nervous system (CNS) diseases has attracted increasing attention [6–8]. Multiple clinical and animal studies have shown the differences in gut microbiota between PD and healthy controls [7, 9–15]. Furthermore, there are increasing numbers of preclinical studies to explore the causal relationship between the gut microbiota and the PD pathogenesis [7, 15, 16]. Short chain fatty acids (SCFAs) are the main gut microbial metabolites that relay signals from the gut microbiota to the host. Accumulating data has shown the beneficial effects of SCFAs on regulating brain function and the integrity of the blood-tissue barrier [17, 18]. However, contradictory to the general recognized beneficial action, one study reported that SCFAs are main regulators accelerating neuroinflammation and α-synucleinopathy in a model of PD [7]. Hence, the search of microbiota or microbiota derived measures to treat or ameliorate PD as an intervening target is a heated topic with many unaddressed question and of our greatest research interest.

The cross-talk between bone-derived osteocalcin (OCN) and brain function is a hot topic [19, 20]. OCN is a marker of bone formation that is specifically secreted by osteoblasts. In recent years, the extraskeletal effects of OCN especially its under-carboxylated form on energy metabolism and brain development have been deeply investigated [20–25]. OCN can pass through the blood-brain barrier, and the peripheral delivery of OCN to aged mice for 2 months can fully restore cognition through direct binding OCN to neurons in the CA3 region of the hippocampus [19, 20]. It is noteworthy that PD patients often have a higher susceptibility to osteoporosis [26], suggesting a link between bone health and PD. Thus, whether OCN could have a protective effect on PD as one of its multiple extra-skeletal benefits is of our research interest.

Additionally, in the process of bone remodeling, the gut microbiota was shown to regulate the activity of osteoblast and osteoclast [27–29]. And a recent finding that the serum level of OCN is associated with the Chao index of gut microbiota species in patients with Crohn’s disease [30] further suggests the potential for OCN to impact microbiota composition. It is thus logical to ask whether there is a link among OCN, the gut microbiota, and PD. Therefore, we hypothesized that OCN can prevent motor impairments and dopaminergic neuronal loss by modulating the gut microbiota in PD mice.

## Results

### OCN administration prevented motor impairments and dopaminergic neuronal loss in 6-OHDA-induced PD mice

**Fig. 1:**
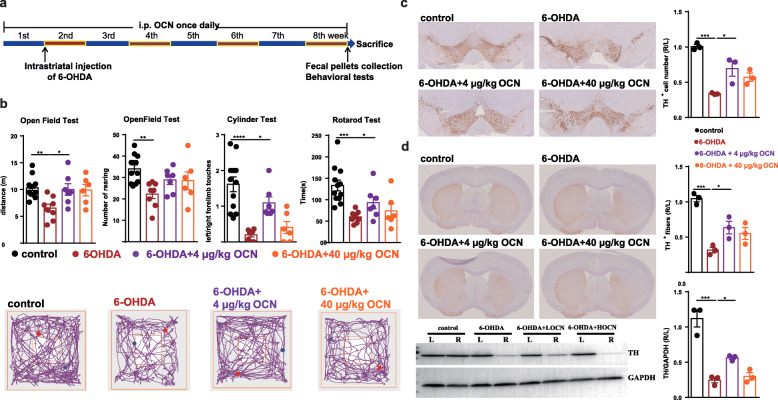
OCN administration prevented motor impairments and dopaminergic neuronal loss in 6-OHDA-induced PD mice. **a** The experimental design of OCN intervention in 6-OHDA-induced PD mice (6-OHDA was injected into the right striatum of the mice). **b** (Upper panel) Bar plots of performance in the behavioral tests, including the open field test, cylinder test and rotarod test. *n* = 6-12 per group. (Lower panel) Representative traces in the open field test. Blue point: starting position; red point: ending position. **c** (Left panel) Representative immunostaining showing TH-positive neurons in the SN. (Right panel) The average number of TH-positive neurons in the ST. *n* = 3 per group, 3 sections per mouse. **d** (Left panel) Representative immunostaining (upper) and western blotting (lower) showing TH-positive fibers and TH protein levels in the striatum. (Right panel) The quantitation of TH-positive fibers (upper) and TH protein levels (lower) in the striatum. Immunostaining: *n* = 3 per group, 3 sections per mouse; western blotting: *n* = 3 per group. LOCN = 4 μg/kg OCN, HOCN = 40 μg/kg OCN. The data represent the mean ± SEM, *p* < 0.05 was set as the threshold for significance by one-way ANOVA followed by post hoc comparisons using Tukey’s test for multiple groups’ comparisons, *p < 0.05, **p < 0.01, ***p < 0.001, ****p < 0.0001

We first examined the effects of the intraperitoneal injection of OCN in a PD mouse model induced by the intrastriatal injection of 6-hydroxydopamine (6-OHDA). 6-OHDA is a hydroxylated analog of the natural dopamine neurotransmitter and used to destroy nigral dopaminergic neurons and deplete the striatum of DA neurotransmitter after the injection into the striatum, thus reproducing the pathological features responsible for motor impairments in PD [31]. Behavioral tests showed significant decreases in movement distance (6-OHDA vs control: *p* = 0.0061) and rearing frequency (6-OHDA vs control: *p* = 0.0042) in the open field test, a defect in frequency of left limb touches in the cylinder test (6-OHDA vs control: *p* < 0.0001), and a decrease in latency in the rotarod test (6-OHDA vs control: *p* = 0.0006) in 6-OHDA-induced PD mice compared with the control group (Fig. 1b, Supplemental Table S1). The administration of 4 μg/kg OCN for 8 weeks significantly improved the motor impairments in the open field test (movement distance, *p* = 0.0343) and cylinder test (the frequency of left limb touches, *p* = 0.0122) in PD mice (Fig. 1b, Supplemental Table S1), but not in control mice (Supplemental Fig. 1A). However, no significant improvements were induced by the administration of 40 μg/kg OCN in 6-OHDA-induced PD mice, suggesting that the dose of OCN is critical for its protective effect in PD (Fig. 1b, Supplemental Table S1).

Given that dopaminergic neuronal loss in the nigrostriatal system is the main pathological characteristics of PD, we examined whether OCN also exerts a protective effect on dopaminergic neurons in the SN and striatum. There was a nearly 70% reduction in tyrosine hydroxylase (TH, the rate limiting enzyme in the synthesis of dopamine and was used as a marker of the dopaminergic neurons) immunostaining, which was used to identify dopaminergic neurons in the SN, on the 6-OHDA-injected side of PD mouse brains (6-OHDA vs control: *p* = 0.0003) compared with control mouse brains, whereas OCN treatment significantly prevented the reduction in dopaminergic neurons in PD mice (6-OHDA vs 6-OHDA+ 4 μg/kg OCN: *p* = 0.0175) (Fig. 1c). Consistent with this observation, immunostaining and western blotting in the striatum also showed remarkable decreases in the TH-positive fibers (6-OHDA vs control: *p* = 0.0003) and TH protein levels (6-OHDA vs control: *p* < 0.0001) on the injected side of striatum of PD mouse brains compared with control mouse brains that were restored after OCN treatment (6-OHDA vs 6-OHDA+ 4 μg/kg OCN: TH-positive fibers, *p* = 0.0471; TH protein levels, *p* = 0.0477) (Fig. 1d). However, there was no significant increase in the number of dopaminergic neurons in the SN and striatum of PD mice after 40 μg/kg OCN administration (Fig. 1c, d). And no changes in the dopaminergic neurons in the SN and striatum of control mice were observed after the intervention of 4 μg/kg OCN (Supplemental Fig. 1B, C). Therefore, this evidence demonstrates that the administration of OCN at a dosage of 4 μg/kg can prevent motor impairments and dopaminergic neuronal loss in 6-OHDA-induced PD mice.

### The gut microbiota mediated the neuroprotective effect of OCN in PD

**Fig. 2:**
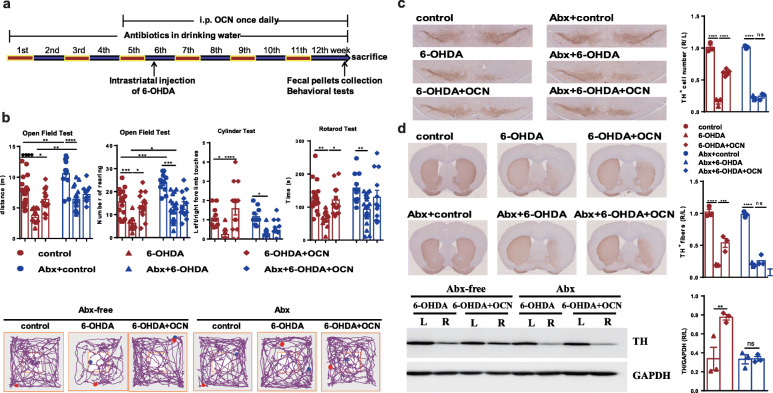
Antibiotic pretreatment (Abx) blocked the OCN-induced improvements of motor impairments and dopaminergic neuronal loss in 6-OHDA-induced PD mice. **a** The experimental design for antibiotic treatment and 4 μg/kg OCN administration in 6-OHDA-induced PD mice. **b** (Upper panel) Bar plots of performance in the behavioral tests including the open field test, cylinder test, and rotarod test. *n* = 11-14 per group. (Lower panel) Representative traces in the open field test. Blue point: starting position; red point: ending position. **c** (Left panel) Representative immunostaining showing TH-positive neurons in the SN. (Right panel) The average number of TH-positive neurons in the ST. *n* = 3 per group, 3 sections per mouse. **d** (Left panel) Representative immunostaining (upper) and western blotting (lower) showing TH-positive fibers and TH protein levels in the striatum. (Right panel) The quantitation of TH-positive fibers (upper) and TH protein levels (lower) in the striatum. Immunostaining: *n* = 3 per group, 3 sections per mouse; western blotting: *n* = 3 per group. The data represent the mean ± SEM, *p* < 0.05 was set as the threshold for significance by one-way ANOVA followed by post hoc comparisons using Tukey’s test for multiple groups’ comparisons, *p < 0.05, **p < 0.01, *** *p* < 0.001, ****p < 0.0001

To test whether the neuroprotective effect of OCN in PD depends on the gut microbiota, we depleted the gut microbiota by using an antibiotic cocktail 4 weeks before OCN administration (Fig. 2a, Supplemental Fig. 2). Gut microbiota-depletion blocked the OCN-induced improvements in motor function, including movement distance (Abx-6-OHDA vs Abx-6-OHDA+OCN: *p* = 0.8932) and number of rearing (Abx-6-OHDA vs Abx-6-OHDA+OCN: *p* = 0.8842) in the open field test, frequency of left limb touches in the cylinder test (Abx-6-OHDA vs Abx-6-OHDA+OCN: *p* = 0.9568), and latency in the rotarod test (Abx-6-OHDA vs Abx-6-OHDA+OCN: *p* = 0.3015) in 6-OHDA-induced PD mice (Fig. 2b, Supplemental Table S2). Notably, gut microbiota depletion significantly increased the motor performance in the open field test (distance: control vs Abx-control: 0.0018, 6-OHDA vs Abx-6-OHDA: *p* = 0.0092; number of rearing: control vs Abx-control: 0.0009, 6-OHDA vs Abx-6-OHDA: 0.0481) (Fig. 2b, Supplemental Table S2).

Further, we examined whether gut microbiota-depletion affects the impact of 6-OHDA and OCN on dopaminergic neuronal loss in PD mice. Immunostaining of TH in the SN showed that 6-OHDA-induced dopaminergic neuronal damage was not affected by gut microbiota-depletion. However, the effect of OCN on preventing the loss of dopaminergic neurons was blocked by gut microbiota-depletion in 6-OHDA-induced PD mice (Abx-6-OHDA vs Abx-6-OHDA+OCN: *p* = 0.9876) (Fig. 2c). The results of immunostaining and western blotting in the striatum confirmed the blocking effect of gut microbiota depletion on OCN-induced protection against dopaminergic neuronal loss (Fig. 2d).

**Fig. 3:**
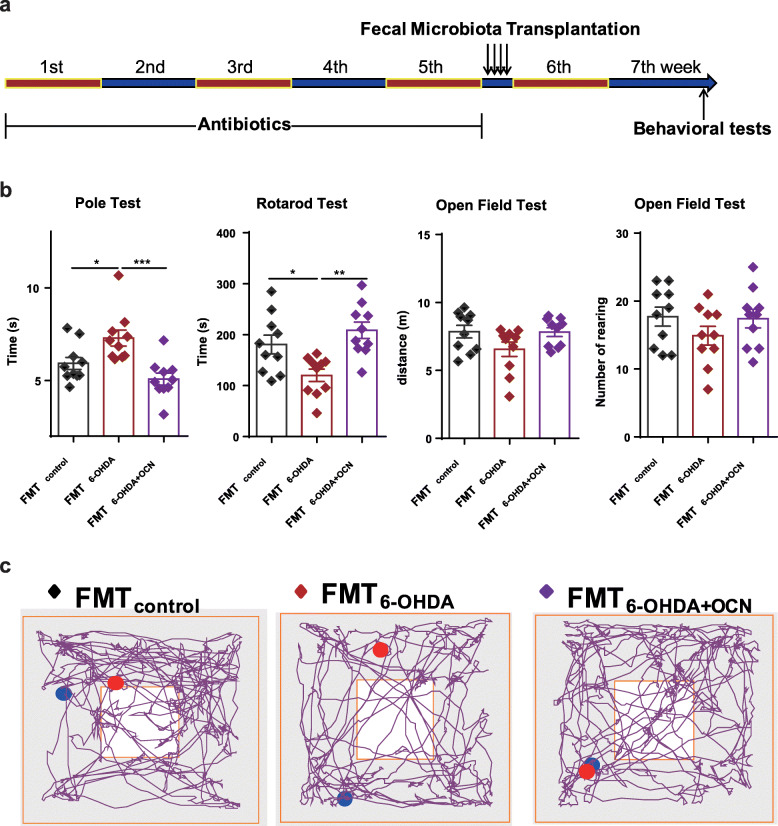
PD-derived microbiota induced PD-like motor impairments in gut microbiota-depleted mice. **a** The experimental design for fecal microbiota transplantation. **b** Bar plots of performance in the behavioral tests including pole test, rotarod test, and open field test. *n* = 10 per group. **c** Representative traces in the open field test. Blue point: starting position; red point: ending position. The data represent the mean ± SEM, *p* < 0.05 was set as the threshold for significance by one-way ANOVA followed by post hoc comparisons using Tukey’s test for multiple groups’ comparisons, *p < 0.05, **p < 0.01, ***p < 0.001, ****p < 0.0001

To further support the notion that gut microbiota alteration mediate the neuroprotective effects of OCN on PD, we transplanted fecal microbiota from control group, 6-OHDA group, and 6-OHDA+OCN group into gut microbiota-depleted mice (which underwent 5 weeks of antibiotic pretreatment). Two weeks after fecal transplantation, we compared the motor function of the different groups of mice (Fig. 3a). In the FMT_6-OHDA_ group, total descent time in the pole test (FMT_control_ vs FMT_6-OHDA_: *p* = 0.0336; FMT_6-OHDA_ vs FMT_6-OHDA + OCN_: *p* = 0.0006) was elongated and latency in the rotarod test (FMT_control_ vs FMT_6-OHDA_: *p* = 0.0288; FMT_6-OHDA_ vs FMT_6-OHDA + OCN_: *p* = 0.0013) was significantly shortened compared to the FMT_control_ and FMT_6-OHDA + OCN_ group (Fig. 3b, Supplemental Table S3). Movement distance and the number of rearings in the open field test were not affected in either FMT_6-OHDA_ or FMT_6-OHDA + OCN_ treated mice (Fig. 3b, Supplemental Table S3). The improved motor performance of mice harboring microbiota from the 6-OHDA+OCN donors compared to the 6-OHDA donors suggests that the gut microbiota might contribute to the benefits conferred by OCN on motor dysfunction in PD mice (Fig. 3b, Supplemental Table S3). Thus, both antibiotic treatment and fecal microbiota transplantation experiments suggest that the gut microbiota is required for the protective effect of OCN in PD.

### Profile of gut microbiota alterations in 6-OHDA-induced PD mice and OCN-treated PD mice

**Fig. 4:**
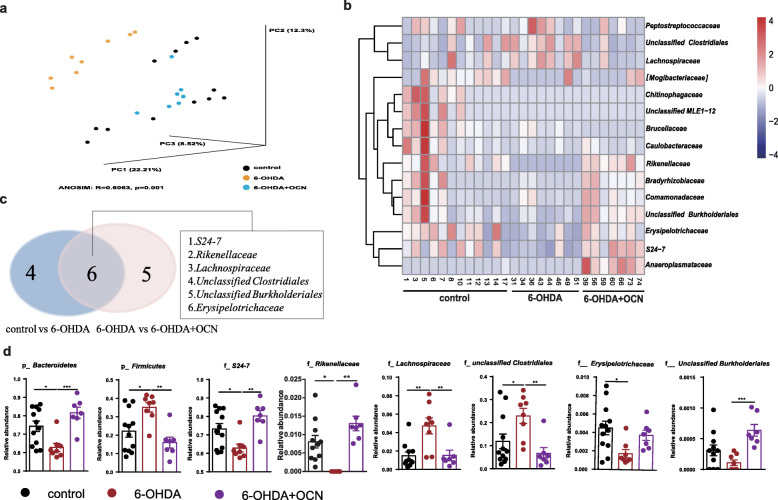
OCN administration modulated gut microbiota dysbiosis in 6-OHDA-induced PD mice. **a** Principal coordinate analysis (PCoA) plot based on unweighted UniFrac distance. **b** Heatmap of the gut microbiota taxa showed different RAs in different groups. Color key represents the RAs. **c** Venn diagram of differential taxa comparisons between control versus 6-OHDA and 6-OHDA+OCN versus 6-OHDA group. **d** Bar plots of the RAs of *p*_*Bacteroidetes* and *p*_*Firmicutes* at the phylum level and of overlapping taxa at the family levels indicated in **c**. The dosage of OCN was 4 μg/kg. *n* = 7–12 per group, data represent the mean ± SEM, *p* < 0.05 was set as the threshold for significance by one-way ANOVA followed by post hoc comparisons using Tukey’s test for multiple groups’ comparisons, *p < 0.05, **p < 0.01, ***p < 0.001, ****p < 0.0001

We next investigated how OCN affected the gut microbiota composition. We performed 16S rRNA sequencing to assess the composition of the gut microbiota. Simpson and Shannon indexes showed no significant differences between groups (Supplemental Fig. 3), suggesting that the diversities of mice gut microbiota were not impacted by 6-OHDA or 4 μg/kg OCN treatment, whereas the UniFrac principal coordinate analysis (PCoA) showed that the gut microbial composition of 6-OHDA-induced PD mice was significantly distinct from that of the control group and that OCN treatment seemed to restore the microbial community structure of PD mice backwards to that of the control group (unweighted ANOSIM: *R* = 0.6063, *p* = 0.001, Fig. 4a). In total, about average 646 OTUs per sample were mapped in phylum level and the mean annotation ratio of family level was 75.39%, while that of genus level was 15.53% (Supplemental Table S4). Thus, we compared the taxonomic alterations mainly in the levels of or above family between groups. Shown in both heatmap and Venn diagram (Fig. 4b, c), we found that six taxa significantly changed in 6-OHDA group vs control group. Specifically, compared with the control group, 6-OHDA-induced PD mice showed a significant decrease in the relative abundances (RAs) of *Bacteroidetes* (6-OHDA vs control: *p* = 0.0176) at the phylum level, *S24-7* (6-OHDA vs control: *p* = 0.0346), *Rikenellaceae* (6-OHDA vs control: *p* = 0.0025), and Erysipelotrichaceae (6-OHDA vs control: *p* = 0.0160) at the family level and a significant increase in the RAs of *Firmicutes* (6-OHDA vs control: *p* = 0.0162) at the phylum level, *Lachnospiraceae* (6-OHDA vs control: *p* = 0.0022), and *unclassified Clostridiales* (6-OHDA vs control: *p* = 0.0455) at the family level. However, OCN administration significantly reversed the changes in PD mice (6-OHDA vs 6-OHDA+OCN: *Bacteroidetes*, *p* = 0.0007 at the phylum level, *S24-7*, *p* = 0.0016; *Rikenellaceae*, *p* < 0.0001 at the family level; *Firmicutes*, *p* = 0.0018 at the phylum level, *Lachnospiraceae*, *p* = 0.0062, *unclassified Clostridiales*, *p* = 0.0083 at the family) (Fig. 4d, Supplemental Table S5). Similar changes were also observed at the genus level (Supplemental Table S5). However, the above alterations in gut microbiota induced by 4 μg/kg OCN treatment was not observed in the control mice (Supplemental Fig. 1D). Together, these data demonstrated the gut microbial features of OCN treatment in PD mice.

**Fig. 5:**
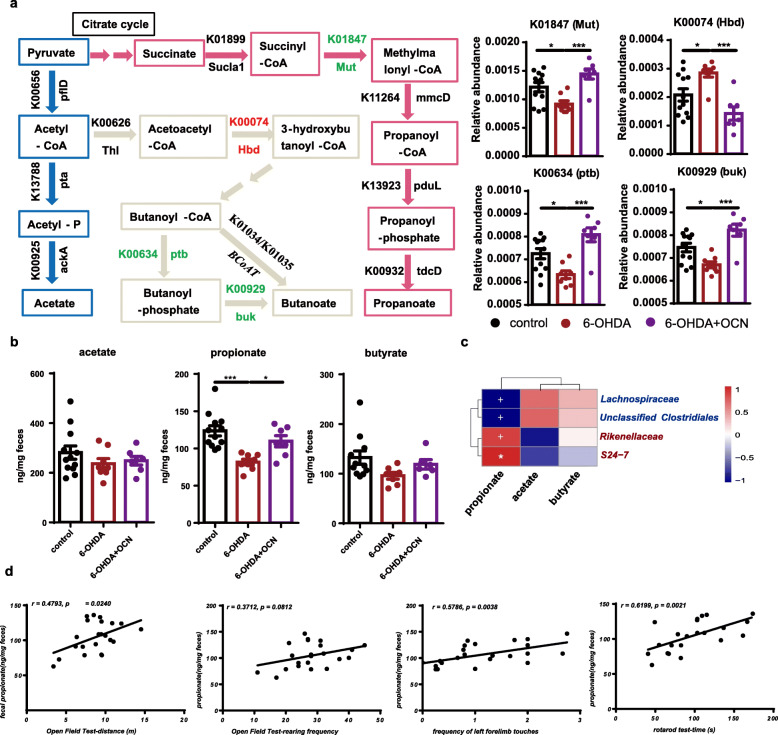
OCN administration increased fecal propionate levels in 6-OHDA-induced PD mice. **a** (Left panel) Metabolic pathway for acetate, propionate, and butyrate synthesis and corresponding KOs and genes are displayed. (Right panel) Bar plots of the RAs of K01847, K00634, K00929, and K00074, *n* = 7–12 per group. **b** Bar plots of comparing the fecal levels of acetate, propionate, and butyrate among groups. Data represent the mean ± SEM, *p < 0.05, **p < 0.01, ***p < 0.001, ****p < 0.0001. **c** Heatmap shows correlations analysis between the fecal level of SCFAs and the RAs of f_*S24-7*, f_*Rikenellaceae*, f_*Lachnospiraceae*, and f_*unclassified Clostridiales*. Color key represents the correlation coefficients, *p < 0.05, ^+^*p* < 0.01. OCN-enriched taxa were marked in red color; depleted taxa were marked in blue. **d** Plots of correlation analysis between the fecal level of propionate and motor function. *X*-axis represented the values of open field test, cylinder test, and rotarod test; *Y*-axis represented the fecal propionate levels. K00656: *pflD*, formate C-acetyltransferase; K13788: *pta*, phosphate acetyltransferase; K00925: *ackA*, acetate kinase; K00626: *Thl*, acetyl-CoA C-acetyltransferase; K00074: *Hbd*, 3-hydroxybutyryl-CoA dehydrogenase; K00634: *ptb*, phosphate butyryltransferase; K00929: *buk*, butyrate kinase; K01034: *but*, acetate CoA-transferase alpha subunit; K01899: *Sucla1*, succinyl-CoA synthetase alpha subunit; K01847: *Mut*, methylmalonyl-CoA mutase; K11264: mmcD, methylmalonyl-CoA decarboxylase; K13923: *pduL*, phosphate propanoyltransferase; K00932: *tdcD*, propionate kinase; K01905: *acdA*, acetate-CoA ligase (ADP-forming) subunit alpha. The dosage of OCN was 4 μg/kg

The Phylogenetic Investigation of Communities by Reconstruction of Unobserved States (PICRUST) was employed to predict the abundance of Kyoto Encyclopedia of Genes and Genomes orthology (KO) from the 16S rRNA gene sequencing data. Compared with that of the control group, the gut microbiome of 6-OHDA-induced PD mice exhibited a significantly decreased potential to produce propionate, as evidenced by the decreased RA of K01847 (methylmalonyl-CoA mutase, *Mut*; 6-OHDA vs control: *p* = 0.0325) (Fig. 5a, Supplemental Table S6). In addition, the RAs of KOs related to butyrate production were altered, but not in the same direction; K00634 (phosphate butyryltransferase, *ptb*; 6-OHDA vs control: *p* = 0.0228) and K00929 (butyrate kinase, *buk*; 6-OHDA vs control: *p* = 0.0277) were significantly decreased and K00074 (3-hydroxybutyryl-CoA dehydrogenase, *Hbd*; 6-OHDA vs control: *p* = 0.0348) was increased, while K01034 and K01035 (the key KOs of rate-limiting enzyme of butyrate production) showed no difference, indicating the unchanged potential in butyrate production (Fig. 5a, Supplemental Table S6). And the RAs of main KOs related to acetate production showed no difference between control and 6-OHDA group (Fig. 5a, Supplemental Table S6). Notably, OCN administration successfully reversed these changes in PD mice (6-OHDA vs 6-OHDA+OCN: K01847, *p* = 0.0008; K00634, *p* = 0.0002; K00929, *p* = 0.0002; K00074, *p* = 0.0006) (Fig. 5a, Supplemental Table S6). However, these KOs of the control mice was not affected by OCN administration (Supplemental Fig. 1E). Therefore, the intervention with OCN can alter the microbiota taxonomy and might increase the bacterial potential of producing propionate in PD mice.

### OCN administration increased fecal propionate levels in 6-OHDA-induced PD mice

The abovementioned results of functional analysis were predictive; to further study whether bacterial SCFAs altered, we assayed the fecal SCFA content by gas chromatography/mass spectrometry (GC/MS). The results revealed a significant decrease in fecal propionate in 6-OHDA-induced PD mice compared with the control group (6-OHDA vs control: *p* = 0.0003), and OCN treatment significantly reversed this change in PD mice (6-OHDA vs 6-OHDA+OCN: *p* = 0.03) (Fig. 5b). There was no significant difference in the fecal levels of acetate and butyrate among the three groups (Fig. 5b). And the fecal level of these three SCFAs in control mice was not affected by OCN administration (Supplemental Fig. 1F). Furthermore, the correlation analysis showed that the fecal propionate level but not the other two SCFAs was positively correlated with the RAs of PD depleted taxa, *S24-7* (*r* = 0.4961, *p* = 0.0010) and *Rikenellaceae* (*r* = 0.5063, *p* = 0.0098) and negatively correlated with the RAs of PD enriched *Lachnosipraceae* (*r* = -0.5843, *p* = 0.0017) and *unclassified Clostridiales* (*r* = -0.5426, *p* = 0.0042) (Fig. 5c), suggesting these microbial changes could be related with the alterations in fecal propionate levels. More importantly, the fecal propionate level was positively correlated with parameters of motor function in the open field test (movement distance: *r* = 0.4973, *p* = 0.0240), cylinder test (frequency of left forelimb touches: *r* = 0.5786, *p* = 0.0038), and rotarod test (latency: *r* = 0.6199, *p* = 0.0021) (Fig. 5d). Thus, the gut microbial mediated neuroprotective effect of OCN might be correlated with its effect on regulating the potential of microbial propionate production which was depleted in gut microbiota of 6-OHDA-induced PD mice.

### Propionate and FFAR3 agonist prevented motor impairments and dopaminergic neuronal loss in 6-OHDA-induced PD mice

**Fig. 6:**
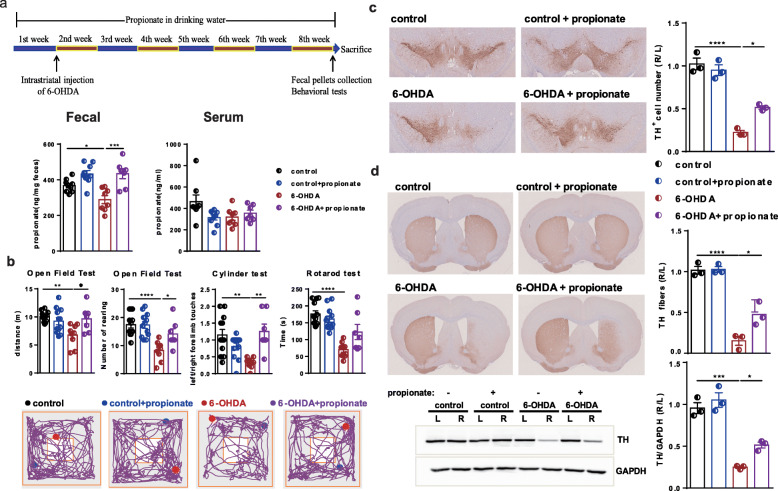
Oral administration of propionate prevented motor impairments and dopaminergic neuronal loss in 6-OHDA-induced PD mice. **a** (Upper panel) The experimental design for propionate intervention in PD mice. (Lower panel) Quantitative measurement by GC/MS showing the fecal and serum levels of propionate. *n* = 7–12 per group. **b** (Upper panel) Bar plots of performance in the behavioral tests, including the open field test, cylinder test, and rotarod test. *n* = 7–12 per group. (Lower panel) Representative traces in the open field test. Blue point: starting position; red point: ending position. **c** (Left panel) Representative immunostaining showing TH-positive neurons in the SN. (Right panel) The average number of TH-positive neurons in the ST. *n* = 3 per group, 3 sections per mouse. **d** (Left panel) Representative immunostaining (upper) and western blotting (lower) showing TH-positive fibers and TH protein levels in the striatum. (Right panel) The quantification of TH-positive fibers (upper) and TH protein levels (lower) in the striatum. Immunostaining: *n* = 3 per group, 3 sections per mouse; western blotting: *n* = 3 per group. The data represent the mean ± SEM, *p* < 0.05 was set as the threshold for significance by one-way ANOVA followed by post hoc comparisons using Tukey’s test for multiple groups’ comparisons, *p < 0.05, **p < 0.01, ***p < 0.001, ****p < 0.0001

To further test the causal relation of propionate with motor function improvements in PD mice, we administered sodium propionate in drinking water to 6-OHDA-induced PD mice for 8 weeks. The intervention with propionate resulted in an obvious increase in fecal propionate levels but not serum propionate levels in 6-OHDA-induced PD mice compared with the control group (Fig. 6a). Importantly, the oral administration of propionate effectively improved motor function in both the open field test (movement distance: 6-OHDA vs 6-OHDA+propionate: *p* = 0.0208; number of rearing: 6-OHDA vs 6-OHDA+propionate: *p* = 0.0339) and cylinder test (6-OHDA vs 6-OHDA+propionate: *p* = 0.0012), but not the rotarod test in 6-OHDA-induced PD mice (Fig. 6b, Supplemental Table S7).

In parallel, the oral administration of propionate prevented nearly 40% of the dopaminergic neuronal loss in the 6-OHDA-injected side of the PD mouse brains (Fig. 6c). The TH protein levels in the striatum induced by propionate also showed a similar pattern of change (Fig. 6d). Thus, we assumed that propionate might be the gut microbiota-derived signal that contributes to PD development and that it may be targeted by OCN for the improvement of this disease.

**Fig. 7:**
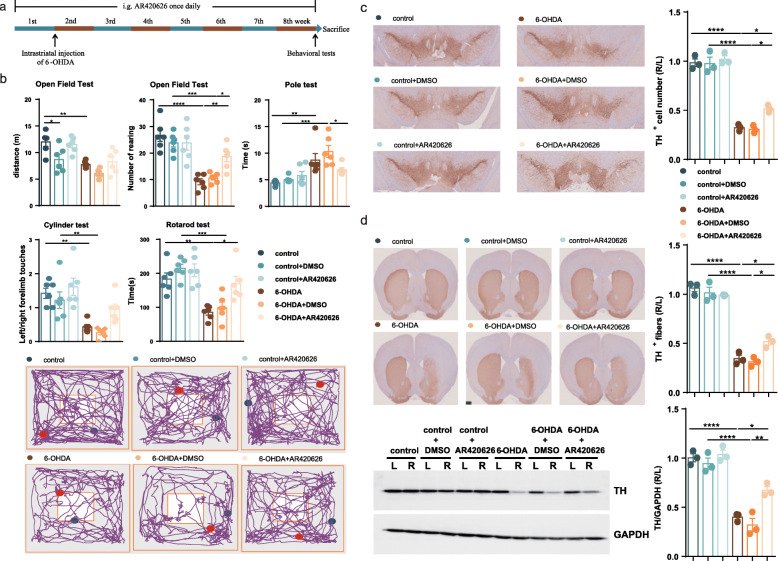
Intragastric administration of FFAR3 agonist prevented motor impairments and dopaminergic neuronal loss in 6-OHDA-induced PD mice. **a** The experimental design for FFAR3 agonist intervention in PD mice. **b** (Upper panel) Bar plots of performance in the behavioral tests, including the open field test, pole test, cylinder test, and rotarod test. *n* = 6 per group. (Lower panel) Representative traces in the open field test. Blue point: starting position; red point: ending position. **c** (Left panel) Representative immunostaining showing TH-positive neurons in the SN. (Right panel) The average number of TH-positive neurons in the ST. *n* = 3 per group, 3 sections per mouse. **d** (Left panel) Representative immunostaining (upper) and western blotting (lower) showing TH-positive fibers and TH protein levels in the striatum. (Right panel) The quantification of TH-positive fibers (upper) and TH protein levels (lower) in the striatum. Immunostaining: *n* = 3 per group, 3 sections per mouse; western blotting: *n* = 3 per group. The data represent the mean ± SEM, *p* < 0.05 was set as the threshold for significance by one-way ANOVA followed by post hoc comparisons using Tukey’s test for multiple groups’ comparisons, *p < 0.05, **p < 0.01, ***p < 0.001, ****p < 0.0001

Since FFAR3 has been reported as the predominant receptor type mediating the protective effect of propionate [18], we next administered FFAR3 agonist (AR420626, 0.1 mg/kg) to 6-OHDA-induced PD mice once daily for 8 weeks by gavage (Fig. 7a). As with the effects of propionate in PD mice, the intervention with AR420626 effectively improved number of rearing in the open field test, but not movement distance, significantly improved motor function in the pole test and rotarod test, but not the cylinder test in 6-OHDA-induced PD mice (Fig. 7b, Supplemental Table S8). In parallel, the intragastric administration of AR420626 prevented 20% of the dopaminergic neuronal loss in the 6-OHDA-injected side of the PD mouse brains (Fig. 7c) and recovered the TH protein levels in the striatum (Fig. 7d). Hence, the agonist of FFAR3, AR420626, mimicked the neuroprotective effects of propionate. And we assumed that gut microbiota-derived propionate might work in part as a FFAR3 agonist to mediate OCN’s protective signal to neuronal system for preventing PD development.

### Enteric nervous system might mediate the neuroprotective effects of propionate in 6-OHDA-induced PD mice

**Fig. 8:**
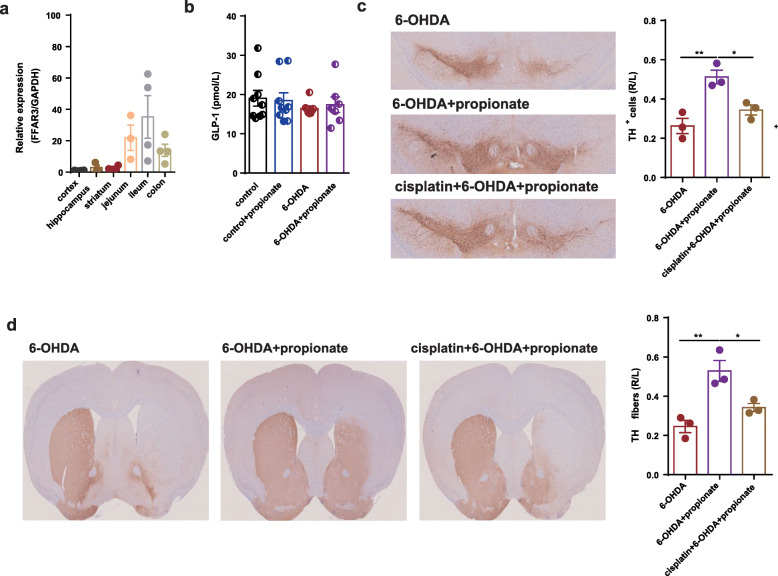
Enteric nervous system mediated the neuroprotective effects of propionate in 6-OHDA-induced PD mice. **a** The relative expression of FFAR3 in cortex, hippocampus, striatum, jejunum, ileum, and colon. *n* = 4. **b** Bar plot of the serum level of Glp-1 among different groups. *n* = 7–12. **c** (Left panel) Representative immunostaining showing TH-positive neurons in the SN. (Right panel) The average number of TH-positive neurons in the ST. *n* = 3 per group, 3 sections per mouse. **d** (Left panel) Representative immunostaining showing TH-positive fibers in the striatum. (Right panel) The quantification of TH-positive fibers in the striatum. Immunostaining: *n* = 3 per group, 3 sections per mouse. The data represent the mean ± SEM, *p* < 0.05 was set as the threshold for significance by one-way ANOVA followed by post hoc comparisons using Tukey’s test for multiple groups’ comparisons, *p < 0.05, **p < 0.01, ***p < 0.001, ****p < 0.0001

Since FFAR3 activation improved motor defects in PD mice, we next asked in which compartment FFAR3 responding to the altered propionate levels dominantly contributed to its neuroprotective effects. Thus, we measured the expression of FFAR3 in different tissues. We found that the relative expression of FFAR3 in the jejunum, ileum, and colon were far higher than that in the neural organs, like the cortex, hippocampus, and striatum (Fig. 8a). Since propionate has been reported to exert its effects by stimulating the release of Glp-1 from enteroendocrine L cells [32] or activating the enteric nervous system (ENS) to transmit its signal to the brain [33], we first measured the serum level of Glp-1 on the basis of the neuroprotective effects of Glp-1 on PD [34–36]. However, no significant difference was observed either between PD and control mice or between propionate treated and untreated PD mice (Fig. 8b). Then, to test whether propionate could act on ENS, we treated 6-OHDA-induced PD mice with cisplatin, a known enteric neurotoxin [37], to deplete PGP9.5-positive enteric cells at the dosage of 3 mg/kg for 4 weeks before the administration of propionate (control vs cisplatin: *p* = 0.0278, Supplemental Fig. 4). In these enteric cells-depleted mice, we found that the effect of propionate to protect dopaminergic neuronal loss was no longer significant (Fig. 8c, d). Thus, we assumed that propionate could work as a FFAR3 agonist targeting ENS to exert the neuroprotective effect in 6-OHDA-induced PD mice.

## Discussion

The finding in our study demonstrated that OCN increased the microbial propionate production, which might activate FFAR3 in enteric neurons, to exert its protective effect on PD. Using a mouse model of 6-OHDA-induced PD, the current study demonstrated that OCN can prevent motor impairments and dopaminergic neuronal loss in PD mice. Our data further suggested that OCN can exert protective effect via regulating microbial metabolism of SCFAs and increasing gut microbiota-derived propionate levels in PD mice. Furthermore, the oral administration of propionate showed protective effect against PD, suggested that microbial propionate production was a potential target for PD intervention. And our data suggested that propionate mainly acted on FFAR3 in enteric neurons to exert its neuroprotective effects on PD.

Multiple clinical and animal studies investigating the correlations between gut microbiome dysbiosis and PD have shown inconsistent observations [10, 14, 15]. The gut microbiota changes found in 6-OHDA-induced PD mice of our study are similar with what have been observed in a study on rotenone-induced PD mouse model [14] demonstrating a decrease in the RAs of *Bacteroidetes* and an increase in the RAs of *Firmicutes* in PD mice, while other studies show the opposite results [10, 13, 15], which had different lengths of intervention and administration route. However, the abovementioned microbial alteration in PD mice in our study can be reversed by OCN administration. In addition, since the effect of OCN in improving motor impairments and dopaminergic neuronal loss in PD mice was attenuated in gut microbiota-depleted mice and the phenotypes observed in PD can be transmitted via fecal transplantation, we proposed that the gut microbiota is required for the neuroprotective effect of OCN in 6-OHDA-induced PD mouse model.

The gut microbiota/brain communication axis has been recently drawn tremendous research attention, whereby the SCFAs, the end products of soluble fiber fermentation by gut microbiota [38], may serve as key signal transmitters. A recent clinical study demonstrated a significant reduction of SCFAs in fecal samples of PD patients compared to healthy controls [11]. Sampson et al. revealed that oral administration of SCFAs to germ-free PD mice promoted neuroinflammation and motor dysfunction [7]. However, in spite of the altered KOs producing both butyrate and propionate in PD mice and recovered by OCN, we only found that fecal propionate was significantly altered, which suggests propionate may dominate the gut microbiota-related effect of OCN on PD in our study. Actually there are 3 KOs related to butyrate production altered in our study, but in the different direction and the corresponding KOs to rate-limiting enzyme of butyrate production showed no difference. In accordance, there are no changes of fecal butyrate assayed by GC/MS. And this finding suggested that butyrate might not be the mediator of the neuroprotective effects of OCN, though butyrate has been suggested to be beneficial to PD [39, 40]. Moreover, the oral propionate replenishment study rescuing the motor impairments and dopaminergic neuronal loss in PD mice further suggested that increased microbial propionate production could play a dominant role in mediating the gut microbiota-related effect of OCN in PD.

Propionate was reported to be mainly produced from succinate by *Bacteroidetes* [41]. Consistent with this finding, our results found a significantly positive correlations between the RAs of *Bacteroidetes*, particularly *S24-7* and *Rikenellaceae* at the family level, with the fecal propionate level. Furthermore, an altered KO (K01847, methylmalonyl-CoA mutase) in the succinate pathway producing propionate was found positively correlated with the RAs of *Bacteroidetes*, *S24-7*, and *Rikenellaceae* (Supplemental Fig. 5), suggesting that these gut microbes may carry the genes encoding methylmalonyl-CoA mutase to regulate succinate pathway of propionate production.

Propionate has been reported to possess multiple beneficial effects on host physiology, such as stimulating intestinal gluconeogenesis [33], shaping the multiple sclerosis disease course by reversing the Treg cell/Th17 cell induction and enhancement of Treg cell function [42] and protecting from hypertensive cardiovascular damage through mitigating the systemic inflammation in hypertension mouse model [43]. However, there also exists some opposite findings, like intraventricular injection of propionate was reported to result in an increase in oxidative stress and microglial activation, involving in some aspects of autism spectrum disorders (ASD) [44]. We found that these inconsistent findings might be due to the different dosage and the route of intervention [45]. Hence, we thought the dosage and administration way might be important to dictate the effect of propionate. In this study, we orally administered 200 mM sodium propionate to mice with decreased level of fecal propionate. After this intervention, the fecal level of propionate in 6-OHDA-induced PD mice increased to the level of control mice. Furthermore, the behavioral results and immunostaining data promoted us to believe the protective effects of propionate on PD, especially on the basis of many latest studies showing the beneficial effects of propionate.

Regarding the underlying mechanism, it has been reported to act primarily through either of the two free fatty acid receptors FFAR2 or FFAR3 [46], widely expressing in multiple organs including the enteroendocrine L cells [32, 47] and the peripheral nervous system [48, 49]. Hence, propionate can exert its effects by stimulating the release of Glp-1 from enteroendocrine L cells or activating the peripheral nervous system to transmit its signal to the brain. Glp-1 has been reported to have clinical benefits in the treatment of moderate PD [34–36], but no significant difference in serum Glp-1 was observed among the groups in our study, hence excluding the possibility of Glp-1 related effect. Next, to further demonstrate the importance of ENS in the regulation of the effect of propionate on PD, the enteric neurotoxin was used to ablate the enteric neurons. The neuroprotective effect of propionate was attenuated after the ablation of enteric neurons, suggesting that ENS might mediate the effect of propionate in 6-OHDA-induced PD mice. FFAR3 was reported as the unique receptor of propionate in the ENS [18], and it has been shown to be activated by propionate to induce IGN via a gut-brain neural circuit [33]. Consistent with these findings, we also showed that FFAR3 might be the sensor of propionate in the ENS since FFAR3 agonist can mimic the neuroprotective effect of propionate in PD mice. Hence, our results suggested that propionate might exert its neuroprotective effects through the activation of FFAR3 in the ENS.

In addition, when the gut microbiota was depleted, PD mice showed significantly better motor performance in the open field test. First, to exclude the possibility that the establishment of PD mouse model was unsuccessful, we evaluated changes in dopaminergic neurons. Even if when the gut microbiota was depleted, the injection of 6-OHDA into the right side of the striatum still caused a large amount of dopaminergic neuronal loss. In fact, other studies have reported similar findings. For example, Diaz et al. reported that germ-free mice display increased motor activity compared with that of mice with a normal gut microbiota [50]. They speculated this behavioral difference is associated with altered expression profiles of canonical signaling pathways, dopamine turnover and synaptic-related proteins in the striatum [50]. In a study on Drosophila, the fly commensal *Lactobacillus brevis*, containing the enzyme xylose isomerase, which modulates sugar metabolism, was found to be responsible for the regulation of locomotor performance [51]. Further work is required to in-depth profile gut microbiome of PD mice and patients, aiming at discovering both taxonomy and functional features for mining the potential microbiota-related pathogenesis and therapeutic targets for PD. Second, from the perspective of the type of behavioral tests, the cylinder test is relatively appropriate to show the importance of gut microbiota in the effect of OCN. The results of cylinder test showed that antibiotic treatment alone cannot reverse the frequency of left limb touches of 6-OHDA-induced PD mice, but blocked the improvement of OCN. Hence, even if Abx alone exerts the effect on motor activities, it cannot affect our current interpretation that Abx might block the effect of OCN on PD.

There are some limitations in our study. First, our study mainly showed the protective effect rather than the therapeutic effect of OCN in PD mice. To further explore the therapeutic effect of OCN administered several weeks after the successful construction of PD model is our research interest in the future. Second, the protective effect of OCN and propionate was not tested in other PD models, like MPTP-induced PD model and rotenone-induced PD model. More in-depth studies and clinical investigations are needed to test and verify our findings.

## Conclusions

In conclusion, the current study indicated that OCN could ameliorate motor deficits and dopaminergic neuronal loss in PD mice, modulating gut microbiome and increasing propionate level could constitute an underlying mechanism responsible for the neuroprotective effects of OCN in PD, and that FFAR3 in the ENS might mediate the neuroprotective effect of propionate in PD.

## Methods

### Chemicals and reagents

The antibodies used in this study were as follows: TH (Sigma Aldrich, AB152) and GAPDH (KeyGEN, KGAA002). The reagents used for western blot were all obtained from Sigma Aldrich. The remaining reagents used in the animal study included osteocalcin (Bachem, H-6552), sodium propionate (Sigma Aldrich, P1880), sodium chloride (Sigma Aldrich, S3014), 6-OHDA (Sigma Aldrich, H116), desipramine (Sigma Aldrich, D3900), ampicillin, neomycin, metronidazole, and vancomycin hydrochloride were all purchased from Sangon Biotech. The BCA kit was obtained from Thermo Fisher Scientific (23259), the QIAamp DNA Stool Mini Kit was purchased from QIAGEN (51504), and the GLP-1 ELISA kit was from Millipore (EGLP-35 K). And the ABC kit and DAB kit were purchased from Vectastain Elite.

### Mice and surgery

All mouse experiments in this study were approved by the ethics committee of Shanghai Jiao Tong University (SJTU). The experiments were conducted according to the National Institutes of Health Guidelines for the Care and Use of Laboratory Animals (NIH publications No. 8023, revised 1978). Male C57BL/6 J mice (23–25 g) were purchased from Beijing Vital River Laboratories (Beijing, China). The mice were housed in a specific-pathogen-free (SPF) environment under controlled conditions, a 12-h light/dark cycle, a temperature of 20–22 °C, and 45 ± 5% humidity, with free access to food and water. All mice were acclimated for one week before the experiment.

A PD model was established by injecting 6-OHDA into the right striatum as previous studies [52–54]. In brief, mice were anesthetized with 1% pentobarbital sodium (0.8 ml/kg) and then placed in a stereotaxic device. After the scalp was incised along the midline, a tiny hole was drilled through the skull above the right striatum according to the following coordinates: AP 0.5 mm, ML 2 mm, and DV 3.3 mm. A total of 10 μg 6-OHDA (5 μg/μl in 2 μl) was injected into the right striatum at a rate of 1 μl/min using a 10-μl Hamilton syringe. The needle was left in place at the injection site for another 10 min and then withdrawn slowly. To prevent 6-OHDA from damaging noradrenergic neurons, an intraperitoneal (i.p.) injection of desipramine (25 mg/kg) was given before 6-OHDA injection.

In the OCN intervention experiment, mice received OCN at one of two doses (4 μg/kg or 40 μg/kg) by intraperitoneal injection once daily for 2 months starting 1 week before the intrastriatal injection of 6-OHDA. The dosages of OCN was determined based on the published study, which showed that mice receiving 3, 10, and 30 ng/h of OCN exhibited a dose-dependent effect in the decrease of fat mass [55]. In this experiment, the mice were randomly divided into five groups (12 mice per group): the control group, in which 0.02% (w/v) ascorbic acid was used as the control for 6-OHDA and normal saline (N.S.) was used as the control for OCN, the control+ 40 μg/kg OCN group, the 6-OHDA group, and the 6-OHDA+OCN (4 μg/kg or 40 μg/kg) group. Seventeen mice died after the surgery. A schematic diagram of the OCN intervention experiment is presented in Fig. 1a.

In the antibiotic treatment experiment, 70 mice were randomly divided into four groups: the 6-OHDA group and the 6-OHDA+OCN group with or without antibiotic treatment. Before the administration of OCN, mice were treated with antibiotics in drinking water for 4 weeks to deplete the gut microbiota. Antibiotic treatment included ampicillin (1 g/L), neomycin (1 g/L), metronidazole (1 g/L), and vancomycin hydrochloride (0.5 g/L). Approximately 99% of fecal bacteria were eliminated by this antibiotic treatment, as has been confirmed in many studies [29, 56–58]. After 1 week of pretreating mice with OCN, 6-OHDA was injected into the right striatum to establish the PD mouse model. Twenty-two mice died after this surgery. Antibiotic treatment lasted for 12 weeks, and OCN treatment lasted for 8 weeks throughout the experiment. A schematic diagram of the antibiotic treatment experiment is shown in Fig. 2a.

In the fecal microbiota transplantation (FMT) experiment, the recipient mice was transplanted by the fecal microbiota from donor mice of the control group (FMT_control_), 6-OHDA group (FMT_6-OHDA_), and 6-OHDA+OCN group (FMT_6-OHDA + OCN_). Fresh fecal pellets were collected from the donors and then diluted immediately with sterile PBS (20 mg fecal pellet/ml). After steeping the stool in sterile PBS for approximately 15 min, it was shaken and centrifuged at 1000 rpm and 4 °C for 5 min. The resulting suspension was centrifuged at 8000 rpm and 4 °C for 5 min to obtain total bacteria and then filtered twice in PBS. The final bacterial suspension was mixed with an equal volume of 40% sterile glycerol to a final concentration of 20% and then stored at − 80 °C until transplantation [59–61]. Mice that received antibiotic treatment for 5 weeks were randomly divided into three groups (10 mice in each group). Then, 100 μl of bacterial suspension was transplanted in each of the recipient mice by gavage. Inoculation was repeated for the next 3 days to reinforce microbiota transplantation. Two weeks later, all mice underwent behavioral tests. A schematic diagram of FMT is shown in Fig. 3a.

In the propionate treatment experiment, mice were provided with drinking water containing sodium propionate (200 mmol/L) or sodium chloride (200 mmol/L) as the control. Forty-eight mice were randomly divided into four groups: the control group, control + propionate group, 6-OHDA group, and 6-OHDA+propionate group. Two-month sodium propionate was administered 1 week before the intrastriatal injection of 6-OHDA. Eight mice died after the injection of 6-OHDA into the striatum. A schematic diagram of this experiment was shown in Fig. 6a.

In the FFAR3 agonist intervention study, FFAR3 agonist, AR420626, was diluted in DMSO solution. Mice received AR420626 (0.1 mg/kg, Sigma, SML1339) intragastrically; DMSO 0.5% in saline was used as vehicle control. Seventy mice were randomly divided into six groups: the control group, control + DMSO group, control + AR420626 group, 6-OHDA group, 6-OHDA + DMSO group, and 6-OHDA + AR420626 group. Two-month AR420626 was administered 1 week before the intrastriatal injection of 6-OHDA. Thirty-four mice died because of the injection of 6-OHDA and intragastric administration. A schematic diagram of this experiment was shown in Fig. 7a.

In the enteric neuropathy study, cisplatin (3 mg/kg, diluted in saline solution, Merck), a known neurotoxin, was administered intraperitoneally weekly, during 4 weeks. After the confirmation of enteric neuropathy by the count of PGP9.5-positive enteric neurons in the ileum, we treated these mice with propionate or sodium chloride for 8 weeks. 6-OHDA was injected into the striatum 1 week after the administration of propionate or sodium chloride.

At the end of these experiments, all mice underwent behavioral tests and were then euthanized. Blood samples were collected and then kept at room temperature for 1 h to ensure complete clotting before centrifugation at 4 °C and 5000 rpm for 10 min to obtain serum samples. Tissues were carefully collected in liquid nitrogen and then stored at − 80 °C until analysis.

### Behavioral tests

Behavioral tests for all animals were performed in the light phase. The open field test was performed to evaluate the motor activity of the mice. The mice were placed in the center of the open field test chamber (a white 27.5 × 27.5 cm chamber with an open top) and allowed to explore freely for 5 min. All activities were recorded by a video camera and tracked using the ANY-maze automated video system. The movement distance and number of rearing were analyzed to evaluate motor function.

A cylinder test was used to evaluate the motor asymmetry of the mice, as unilateral 6-OHDA-induced lesions in the right striatum cause motor dysfunction of the left limbs. Each mouse was placed into a transparent cylinder (25 cm in diameter, 60 cm high) for 3 min, and the use of each forelimb during rearing was recorded by an assessor who was blind to the treatment conditions. The data are presented as the frequency ratio of left/right forelimb wall touches.

A rotarod test was also performed to evaluate the motor function of the mice. Briefly, mice were placed on an accelerating rod with 5 individual chambers. The rotation speed of the rod started at 4 rpm and gradually increased to 40 rpm. The latency to fall from the rod was recorded for a maximum of 300 s. Five trials were repeated, and the average latency was used for statistical analyses.

The pole test was used in the FMT experiment instead of cylinder test as there were no unilateral lesions-induced in this experiment. A 0.5-m long, 1-cm-diameter pole that had a 2-cm-diameter spherical protuberance on top and was wrapped with non-adhesive gauze to facilitate gripping was placed in the home cage. Timing began when the experimenter released the mouse and ended when one hind limb reached the home cage base. The total time required to climb down the pole was measured. Each mouse performed 3 successive trials. The average of the three trials was calculated for statistical analyses.

### Western blotting

Mouse brain tissues were lysed in RIPA buffer with 1× protease and phosphatase inhibitor cocktail to extract total protein. The total protein concentration in the supernatant was determined by a BCA Kit. After the supernatant was denatured by boiling for 5 min in loading buffer, equal amounts of proteins were run on a 10% SDS-PAGE gel and then transferred onto a PVDF membrane. The membranes were blocked with 5% nonfat milk in 0.1% TBST for 2 h at room temperature and then incubated with a primary antibody overnight at 4 °C. After extensive washing, the membranes were incubated with the appropriate HRP-conjugated secondary antibody at room temperature for 2 h. Finally, the blots were developed with an ECL reagent. The following primary antibodies were used: the antibody against tyrosine hydroxylase (TH, 1:10000) and anti-GAPDH (1:20000). The densitometry analysis of the band of TH protein was analyzed using ImageJ 1.49v (National Institutes of Health, USA, https://imagej.en.softonic.com/).

### Immunochemistry

After behavioral tests, 3-5 mice per group were anesthetized with 1% pentobarbital sodium (0.8 ml/kg), perfused transcardially with PBS buffer, and fixed with 4% paraformaldehyde (PFA) fixative solution. The brains and ileum were dissected, fixed, dehydrated, embedded in paraffin, and sectioned at a thickness of 4 μm. Every fifth section containing the SN or striatum was collected, and 3 sections per sample were subjected to immunostaining. Briefly, after blocking endogenous peroxidase activity with 3% H_2_O_2_ and antigen retrieval with citric acid buffer, the sections were blocked with 5% goat serum and incubated with a primary antibody against TH (1:400) for brains and PGP9.5 (1:2000) for ileum overnight at 4 °C. On the second day, the sections were incubated with a biotin-conjugated goat anti-rabbit IgG antibody, and staining was performed using the avidin-biotin complex (ABC) system and nickel-enhanced diaminobenzidine (DAB) incubation. Images were obtained using a microscope (Olympus IX70, Japan). The number of TH-positive neurons in the SN and PGP9.5-positive enteric neurons and the fractions of the TH-positive area in the striatum were measured using ImageJ. The data are expressed as a percentage of the number of TH-positive neurons or area fraction on the impaired side relative to that on the intact side.

### Fecal sample collection

To collect fecal pellets, mice were placed individually in empty autoclaved cages and allowed to defecate freely from 7 am to 10 am 3 days before the behavioral tests. Once feces were formed, they were collected immediately in individual sterile tubes at − 20 °C and then stored at − 80 °C.

### Stool culture measurement

0.2 g fresh fecal samples were collected in pre-weighted tubes containing 1 mL phosphate-buffered saline (PBS). Samples were thoroughly vortexed and diluted with PBS to 1:10000; samples from antibiotic-treated mice were collected 4 and 5 weeks after antibiotic initiation. Equal concentrations of stool diluent from antibiotic, control, and blanks were placed on LB agar plates and incubated for 24 h.

### Gut microbe 16S rRNA sequencing and data analyzing

Microbial genomic DNA was extracted from fecal samples by a QIAamp DNA Stool Mini Kit following the manufacturer’s protocol. To ensure successful DNA isolation, the quantity of the DNA was measured using a NanoDrop instrument (Thermo Fisher Scientific, Waltham, MA, USA), the quality of the DNA was then assessed by agarose gel electrophoresis and the sterile water was used as the negative control.

The forward primer (338F 5′-ACTCCTACGGGAGGCAGCA-3′) and the reverse primer (806R 5′-GGACTACHVGGGTWTCTAAT -3′) were used to amplify the V3-V4 regions of the 16S rRNA gene. The 16S rRNA gene amplicon and sequencing were performed as described previously [62]. The sequencing data has been deposited in the Sequence Read Archive (SRA) of National Center for Biotechnology Information (NCBI) (Bioproject: PRJNA613781) to be released upon publication.

16S rRNA gene sequencing analysis was performed using QIIME (Quantitative Insights Into Microbial Ecology, v1.8.0, http://qiime.org/) and the R package 3.5.1 (https://www.r-project.org/). Beta diversity analysis was performed to investigate the structural variation of microbial communities across samples using UniFrac distance metrics and visualized via principal coordinate analysis (PCoA). Differences in the UniFrac distances among groups were determined by analysis of similarities (ANOSIM). LEfSe was performed to detect differentially abundant taxa at the phylum, family, and genus levels across groups using the default parameters. Heatmap was plotted using R package 3.5.1.

Phylogenetic Investigation of Communities by Reconstruction of Unobserved States (PICRUSt) based on OTUs was employed to predict the abundances of functional categories using Kyoto Encyclopedia of Genes and Genomes (KEGG) orthologs (KO). The relative abundance of the KO associated with the metabolism of SCFAs was further analyzed by one-way ANOVA.

### Fecal short-chain fatty acid (SCFA)

Fecal SCFA concentrations were measured by gas chromatography/mass spectrometry (GC/MS). Briefly, 0.1 g of feces were homogenized with 0.4 ml ddH2O and centrifuged at 4 °C and 5000 rpm for 5 min. Aliquots (0.2 ml) of the supernatants were collected, and then 0.05 ml of 50% H_2_SO_4_ and 0.25 ml of diethyl ether containing 50 μg/ml 2-methylpentanoic acid were added. After vortexing for 2 min, the mixture was centrifuged at 4 °C and 12000 rpm for another 10 min. After incubation at − 20 °C for 30 min, the supernatants were collected in a vial containing anhydrous sodium sulfate and then measured by GC on an Agilent7890B-7000D system (Agilent Technologies, CA, USA) equipped with flame ionization, thermal conductivity detectors, capillary columns, and GC ChemStation software. Acetate, propionate, and butyrate were quantified using pure standards diluted in diethyl ether.

### Glp-1 analysis

The serum level of glucagon-like peptide (Glp-1) was measured by an ELISA kit (Millipore EGLP-35k) following the manufacturer’s protocol. Before the collection of blood samples, 10 μl of DPP-IV inhibitor was added to the tubes to inhibit the degradation of Glp-1.

### Statistical analysis

Except for the 16S rRNA data has been described in detail. Data obtained in this study were analyzed using GraphPad Prism 6 software (https://www.graphpad.com/) by one-way ANOVA followed by post hoc comparisons using Tukey’s test for multiple group comparisons. Spearman correlation analyses were used for 16S rRNA data, and the correlation between fecal propionate level and motor function were analyzed by Pearson correlation analyses. The data are presented as the mean ± SEM, and *p* < 0.05 was set as the threshold for significance.

### Supplementary Information

**Additional file 1: Supplemental Table S1.** The comparison of the behavioral tests in 6-OHDA-induced PD mice after the administration of OCN. **Supplemental Table S2.** The comparison of the behavioral tests in 6-OHDA-induced PD mice treated with OCN after gut microbiota-depletion. **Supplemental Table S3.** The comparison of the behavioral tests after fecal microbiota transplantation. **Supplemental Table S4.** The overall OTU numbers and annotation levels. **Supplemental Table S5.** The comparison of taxonomy RAs of gut microbiota in 6-OHDA-induced mice after the administration of OCN. **Supplemental Table S6.** The comparison of RAs of KOs regulating SCFA metabolism in 6-OHDA-induced mice after the administration of OCN. **Supplemental Table S7.** The comparison of the behavioral tests in 6-OHDA-induced mice after the administration of propionate. **Supplemental Table S8.** The comparison of the behavioral tests in 6-OHDA-induced mice after the administration of FFAR3 agonist. **Supplemental Figure 1.** OCN administration had no effect on motor function, dopaminergic neurons, gut microbiota and SCFAs. **Supplemental Figure 2.** Antibiotic treatment depleted the gut microbiota. **Supplemental Figure 3.** The comparison of alpha diversity of gut microbiota in 6-OHDA-induced PD mice. **Supplemental Figure 4.** Cisplatin ablated the enteric neurons. **Supplemental Figure 5.** Correlation analysis between the altered KO and gut microbial taxa.

## Data Availability

The 16S rRNA sequencing data has been deposited in the Sequence Read Archive (SRA) of National Center for Biotechnology Information (NCBI) (Bioproject: PRJNA613781) to be released upon publication. The data generated or analyzed during this study are included in this article and its supplemental information files.

## Abbreviations

Abx: Antibiotic treatment
BBB: Blood brain barrier
ENS: Enteric nervous system
FMT: Fecal microbiota transplantation
FFAR: Free fatty acid receptor
GC/MS: Gas chromatography/mass spectrometry
GLP-1: Glucagon-like peptide 1
OCN: Osteocalcin
OTU: Operational taxonomic unit
PD: Parkinson’s disease
RAs: Relative abundances
TH: Tyrosine hydroxylase
TLR4: Toll-like receptor 4
6-OHDA: 6-Hydroxydopamine
