# Correction: Gut microbiota-derived propionate mediates the neuroprotective effect of osteocalcin in a mouse model of Parkinson’s disease

**DOI:** 10.1186/s40168-024-01846-5

**Published:** 2024-06-21

**Authors:** Yan-fang Hou, Chang Shan, Si-yue Zhuang, Qian-qian Zhuang, Arijit Ghosh, Ke-cheng Zhu, Xiao-ke Kong, Shu-min Wang, Yan-ling Gong, Yu-ying Yang, Bei Tao, Li-hao Sun, Hong-Yan Zhao, Xing-zhi Guo, Wei-qing Wang, Guang Ning, Yan-yun Gu, Sheng-tian Li, Jian-min Liu

**Affiliations:** 1 Department of Endocrine and Metabolic Diseases, Ruijin Hospital, Shanghai Jiao Tong University School of Medicine, Shanghai Institute of Endocrine and Metabolic Diseases, Shanghai Clinical Center for Endocrine and Metabolic Diseases, Shanghai, 200025 China; 2 Bio-X Institutes, Key Laboratory for the Genetics of Developmental and Neuropsychiatric Disorders (Ministry of Education), Shanghai Key Laboratory of Psychotic Disorders, and Brain Science and Technology Research Center, Shanghai Jiao Tong University, Shanghai, 200240 China

**Correction** **:** **Microbiome 9, 34 (2021)**

**https://doi.org/10.1186/s40168-020-00988-6**

Following publication of the original article [1], it was reported that the Supplementary Material did not include the Supplementary Figures. The correct Supplementary file is included here and the original article has been updated.

### Supplementary Information

Additional file 1: Supplemental Table S1. The comparison of the behavioral tests in 6-OHDA-induced PD mice after the administration of OCN. Supplemental Table S2. The comparison of the behavioral tests in 6-OHDA-induced PD mice treated with OCN after gut microbiota-depletion. Supplemental Table S3. The comparison of the behavioral tests after fecal microbiota transplantation. Supplemental Table S4. The overall OTU numbers and annotation levels. Supplemental Table S5. The comparison of taxonomy RAs of gut microbiota in 6-OHDA-induced mice after the administration of OCN. Supplemental Table S6. The comparison of RAs of KOs regulating SCFA metabolism in 6-OHDA-induced mice after the administration of OCN. Supplemental Table S7. The comparison of the behavioral tests in 6-OHDA-induced mice after the administration of propionate. Supplemental Table S8. The comparison of the behavioral tests in 6-OHDA-induced mice after the administration of FFAR3 agonist. Supplemental Figure 1. OCN administration had no effect on motor function, dopaminergic neurons, gut microbiota and SCFAs. Supplemental Figure 2. Antibiotic treatment depleted the gut microbiota. Supplemental Figure 3. The comparison of alpha diversity of gut microbiota in 6-OHDA-induced PD mice. Supplemental Figure 4. Cisplatin ablated the enteric neurons. Supplemental Figure 5. Correlation analysis between the altered KO and gut microbial taxa.
