# Supplementary material for: Gut microbiota-derived propionate mediates the neuroprotective effect of osteocalcin in a mouse model of Parkinson’s disease
Source: Microbiome. 2021 Jan 31;9:34. doi: 10.1186/s40168-020-00988-6 (PMC7849090; doi:10.1186/s40168-020-00988-6)

Gut microbiota-derived propionate mediates the neuroprotective effect of osteocalcin in a mouse model of Parkinson's disease

Yan-fang Hou[1], Chang Shan[1], Arijit Ghosh[2], Qian-qian Zhuang[2], Shu-min Wang[1], Si-yue Zhuang[2], Yan-ling Gong[2], Qian Wang[2], Yu-ying Yang[1], Bei Tao[1], Li-hao Sun[1], Hong-Yan Zhao[1], Xing-zhi Guo[1], Wei-qing Wang[1], Guang Ning[1], Yan-yun Gu[1,*], Sheng-tian Li[2,*], Jian-min Liu[1,3,*]

[1]Department of Endocrine and Metabolic Diseases, Rui-jin Hospital, Shanghai Jiao Tong University School of Medicine, Shanghai Institute of Endocrine and Metabolic Diseases, Shanghai Clinical Center for Endocrine and Metabolic Diseases, Shanghai, 200025, China.
[2]Bio-X Institutes, Key Laboratory for the Genetics of Developmental and Neuropsychiatric Disorders (Ministry of Education), Shanghai Key Laboratory of Psychotic Disorders, and Brain Science and Technology Research Center, Shanghai Jiao Tong University, Shanghai, 200240, China
[3]Lead contact
*Correspondence: guyanyun@hotmail.com (Y.G.), lstian@sjtu.edu.cn (S.L.), ljm10586@rjh.com.cn (J.L.)

Supplemental Table S1 The comparison of the behavioral tests in 6-OHDA-induced PD mice after the administration of OCN.

Supplemental Table S2 The comparison of the behavioral tests in 6-OHDA-induced PD mice treated with OCN after gut microbiota-depletion.

Supplemental Table S3 The comparison of the behavioral tests after fecal microbiota transplantation.

Supplemental Table S4 The overall OTU numbers and annotation levels.

Supplemental Table S5 The comparison of taxonomy RAs of gut microbiota in 6-OHDA-induced mice after the administration of OCN.

Supplemental Table S6 The comparison of RAs of KOs regulating SCFA metabolism in 6-OHDA-induced mice after the administration of OCN.

Supplemental Table S7 The comparison of the behavioral tests in 6-OHDA-induced mice after the administration of propionate.

Supplemental Table S8 The comparison of the behavioral tests in 6-OHDA-induced mice after the administration of FFAR3 agonist.

Supplemental Fig. 1 OCN administration had no effect on motor function, dopaminergic neurons, gut microbiota and SCFAs.

Supplemental Figure. 2 Antibiotic treatment depleted the gut microbiota.

Supplemental Figure. 3 The comparison of alpha diversity of gut microbiota in 6-OHDA-induced PD mice.

Supplemental Figure. 4 Cisplatin ablated the enteric neurons.

Supplemental Fig. 5 Correlation analysis between the altered KO and gut microbial taxa.

Supplemental Table S1 The comparison of the behavioral tests in 6-OHDA-induced PD mice after the administration of OCN.

Behavioral Tests	control (A, n = 12)	6-OHDA (B, n = 8)	6-OHDA+ 4 μg/kg OCN (C, n = 7)	6-OHDA+ 40 μg/kg OCN (D, n = 6)	P value Total	A vs B	B vs C	B vs D
**Open Field Test**								
Distance (m)	10.32 ± 0.56	6.52 ± 0.75	9.99 ± 1.08	9.88 ± 1.06	0.0068	0.0061	0.0343	0.0539
Number of rearing	34.08 ± 1.82	22.25 ± 2.09	28.86 ± 2.11	28.50 ± 4.16	0.0085	0.0042	0.2737	0.3561
**Cylinder test**								
Left/right forelimb touches	1.61 ± 0.21	0.19 ± 0.05	1.08 ± 0.17	0.40 ± 0.17	< 0.0001	< 0.0001	0.0122	0.8800
**Rotarod test**								
Time (s)	133.10 ± 12.44	59.63 ± 4.82	92.90 ± 13.81	73.94 ± 16.16	0.0006	0.0006	0.2999	0.8815

Data represented mean ± SEM, $p < 0.05$ was set as the threshold for significance by one-way ANOVA followed by post hoc comparisons using Tukey's test for multiple groups' comparisons.

Supplemental Table S2 The comparisons of the behavioral tests in 6-OHDA-induced PD mice treated with OCN after gut microbiota-depletion.

Behavioral Tests	control	6-OHDA	6-OHDA+OCN	Abx+control	Abx+6-OHDA	Abx+6-OHDA+OCN	P value			
	(A, n = 18)	(B, n = 11)	(C, n = 14)	(D, n = 11)	(E, n = 20)	(F, n = 12)	Total	A vs D	B vs C	E vs F
**Open Field Test**										
Distance (m)	7.57 ± 0.58	3.79 ± 0.35	6.35 ± 0.51	10.60 ± 0.77	6.69 ± 0.79	7.18 ± 0.42	<0.0001	0.0018	0.0218	0.8932
Number of rearing	15.50 ± 1.33	6.27 ± 0.90	13.93 ± 1.46	23.82 ± 1.21	10.00 ± 1.64	14.00 ± 1.65	<0.0001	0.0009	0.0053	0.8842
**Cylinder test**										
Left/right forelimb touches	1.08 ± 0.09	0.23 ± 0.11	1.57 ± 0.32	1.16 ± 0.14	0.25 ± 0.11	0.46 ± 0.15	< 0.0001	0.9995	<0.0001	0.9568
**Rotarod test**										
Time (s)	129.30 ± 10.65	65.36 ± 7.51	121.30 ± 11.32	157.00 ± 13.44	93.47 ± 15.56	129.6 ± 20.98	0.0002	0.6426	0.0471	0.3015

The dose of OCN was 4 μg/kg. Data represented mean ± SEM, $p < 0.05$ was set as the threshold for significance by one-way ANOVA followed by post hoc comparisons using Tukey's test for multiple groups' comparisons.

Supplemental Table S3 The comparisons of the behavioral tests after fecal microbiota transplantation.

Behavioral Tests	$FMT_{control}$	$FMT_{6\text{-}OHDA}$	$FMT_{6\text{-}OHDA+OCN}$	P value		
	(A, n = 10)	(B, n = 10)	(C, n = 10)	Total	A vs B	B vs C
**Open Field Test**						
Distance (m)	7.85 ± 0.46	6.55 ± 0.53	7.81 ± 0.33	0.0854	0.1217	0.1361
Number of rearing	17.70 ± 1.39	14.90 ± 1.36	17.40 ± 1.36	0.3005	0.3331	0.4131
**Pole Test**						
Time (s)	5.92 ± 0.33	7.29 ± 0.43	5.08 ± 0.32	0.0008	0.0336	0.0006
**Rotarod test**						
Time (s)	180.80 ± 18.30	120.20 ± 12.12	208.60 ± 16.06	0.0016	0.0288	0.0013

Data represented mean ± SEM, $p < 0.05$ was set as the threshold for significance by one-way ANOVA followed by post hoc comparisons using Tukey's test for multiple groups' comparisons.

Supplemental Table S4 The overall OTU number and annotation levels.

	Annotated OTUs(n)	Annotation rate		
		Order	Family	Genus
control	669.1 ± 137.3	100%	77.06%	15.81%
6-OHDA	732.4 ± 80.82	100%	65.65%	16.13%
6-OHDA+OCN	509.3 ± 75.55	100%	87.63%	13.97%
Total	646.4 ± 136.50	100%	75.39%	15.53%

Data represented mean ± SD.

Supplemental Table S5 The comparison of taxonomy RAs of gut microbiota in 6-OHDA-induced mice after the administration of OCN.

Bacteria	control (A, n = 12)	6-OHDA (B, n = 8)	6-OHDA+OCN (C, n = 7)	P value Total	A vs B	B vs C
**Phylum**						
Bacteroidetes	0.7429 ± 0.0284	0.6286 ± 0.0215	0.8160 ± 0.0302	0.0009	0.0176	0.0007
Firmicutes	0.2219 ± 0.0324	0.3513 ± 0.0258	0.1597 ± 0.0298	0.0018	0.0162	0.0018
**Family**						
S24-7	0.7323 ± 0.0290	0.6286 ± 0.0215	0.8030 ± 0.0295	0.0021	0.0346	0.0016
unclassified Clostridiales	0.1188 ± 0.0316	0.2292 ± 0.0329	0.0669 ± 0.0244	0.0084	0.0455	0.0083
Lachnospiraceae	0.0148 ± 0.0041	0.0472 ± 0.0086	0.0152 ± 0.0057	0.0014	0.0022	0.0062
Rikenellaceae	0.0081 ± 0.0017	6.338e-006 ± 6.338e-006	0.0130 ± 0.0020	< 0.0001	0.0025	< 0.0001
Erysipelotrichaceae	0.0045 ±0.0007	0.0017 ± 0.0004	0.0038 ± 0.0006	0.0199	0.0160	0.1432
**Genus**						
unclassified S24-7	0.7323 ± 0.0290	0.6286 ± 0.0215	0.8030 ± 0.0295	0.0021	0.0346	0.0016
unclassified Clostridiales	0.1188 ± 0.0316	0.2292 ± 0.0329	0.0669 ± 0.0244	0.0084	0.0455	0.0083
unclassified Lachnospiraceae	0.0113 ± 0.0034	0.0396 ± 0.0079	0.0107 ± 0.0049	0.0013	0.0023	0.0052
unclassified Rikenellaceae	0.0081 ± 0.0017	6.338e-006 ± 6.338e-006	0.0130 ± 0.0020	< 0.0001	0.0025	< 0.0001

Data represented mean ± SEM, $p < 0.05$ was set as the threshold for significance by one-way ANOVA followed by post hoc comparisons using Tukey’s test for multiple groups’ comparisons. RAs = relative abundances.

Supplemental Table S6 The comparison of RAs of KOs regulating SCFA metabolism in 6-OHDA-induced mice after the administration of OCN.

KO	control (A, n = 12)	6-OHDA (B, n = 8)	6-OHDA+OCN (C, n = 7)	P value Total	A vs B	B vs C
K00656	0.001183 ± 5.093e-005	0.001332 ± 4.769e-005	0.001095 ± 4.546e-005	0.0184	0.1011	0.0161
K13788	1.459e-005 ± 2.445e-006	1.111e-005 ± 4.989e-006	8.693e-006 ± 1.472e-006	0.0436	0.7163	0.8815
K00925	0.0009388 ± 6.245e-006	0.0009353 ± 7.379e-006	0.0009581 ± 7.176e-006	0.0908	0.9246	0.1051
K01847	0.001214 ± 8.205e-005	0.0009171 ± 5.667e-005	0.001445 ± 8.812e-005	0.0011	0.0325	0.0008
K11264	1.028e-005 ± 1.717e-006	9.405e-006 ± 4.641e-006	5.917e-006 ± 9.615e-007	0.5355	0.9705	0.6964
K13923	1.050e-005 ± 1.751e-006	9.521e-006 ± 4.690e-006	5.937e-006 ± 9.542e-007	0.5166	0.9645	0.6891
K00932	9.578e-006 ± 1.991e-006	4.385e-006 ± 1.496e-006	5.551e-006 ± 3.432e-007	0.0849	0.0951	0.9027
K00626	0.0002503 ± 2.372e-005	0.0003044 ± 8.150e-006	0.0001684 ± 2.307e-005	0.0018	0.1802	0.0013
K00074	0.0002078 ± 2.159e-005	0.0002846 ± 1.425e-005	0.0001422 ± 2.332e-005	0.0009	0.0348	0.0006
K01692	0.0001377 ± 3.431e-005	0.0001150 ± 2.087e-005	0.0001240 ± 2.330e-005	0.8592	0.8521	0.9804
K00634	0.0007249 ± 2.221e-005	0.0006325 ± 1.705e-005	0.0008077 ± 3.053e-005	0.0003	0.0228	0.0002
K00929	0.0007458 ± 1.887e-005	0.0006698 ± 1.339e-005	0.0008219 ± 2.657e-005	0.0002	0.0277	0.0002
K01034	4.903e-005±9.392e-006	5.426e-005±1.323e-005	4.274e-005±6.266e-006	0.7751	0.9278	0.7556
K01035	6.993e-006±1.388e-006	3.380e-006±1.175e-006	3.389e-006±6.149e-006	0.0689	0.1154	>0.9999

Data represented mean ± SEM, $p < 0.05$ was set as the threshold for significance by one-way ANOVA followed by post hoc comparisons using Tukey's test for multiple groups' comparisons. RAs = relative abundances.

Supplemental Table S7 The comparison of the behavioral tests in 6-OHDA-induced mice after the administration of propionate.

Behavioral Tests	control (A, n = 12)	control+PPA (B, n = 12)	6-OHDA (C, n = 9)	6-OHDA+PPA (D, n = 7)	P value Total	A vs C	C vs D
**Open Field Test**							
Distance (m)	10.05 ± 0.27	9.23 ± 0.63	6.77 ± 0.65	9.64 ± 0.88	0.0022	0.0017	0.0208
Number of rearing	17.42 ± 1.17	17.33 ± 1.21	8.22 ± 1.15	14.14 ± 1.82	< 0.0001	< 0.0001	0.0339
**Cylinder test**							
Left/right forelimb touches	1.15 ± 0.17	0.80 ± 0.10	0.33 ± 0.06	1.25 ± 0.22	0.0004	0.0011	0.0012
**Rotarod test**							
Time (s)	175.60 ± 9.88	160.70 ± 9.28	69.93 ± 7.35	122.3 ± 22.5	0.0001	< 0.0001	0.5025

PPA = propionate, Data represented mean ± SEM, $p < 0.05$ was set as the threshold for significance by one-way ANOVA followed by post hoc comparisons using Tukey’s test for multiple groups’ comparisons.

Supplemental Table S8 The comparison of the behavioral tests in 6-OHDA-induced mice after the administration of FFAR3 agonist.

Behavioral Tests	control (A, n = 6)	control+DMSO (B, n = 6)	control +AR420626 (C, n = 6)	6-OHDA (D, n = 6)	6-OHDA+ DMSO (E, n = 6)	6-OHDA+ AR420626 (F, n = 6)	P value Total	B vs E	E vs F
**Open Field Test**									
Distance (m)	11.98 ± 0.83	8.69 ± 0.98	11.48 ± 0.59	7.74 ± 0.27	6.09 ± 0.33	8.28 ± 0.92	<0.0001	0.1299	0.2751
Number of rearing	26.67 ± 2.25	23.83 ± 1.58	23.83 ± 2.74	9.17 ± 1.14	10.67 ± 0.76	18.67 ± 1.87	<0.0001	0.0003	0.0472
**Cylinder test**									
Left/right forelimb touches	1.42 ± 0.15	1.22 ± 0.24	1.61 ± 0.26	0.43 ± 0.07	0.26 ± 0.06	0.94 ± 0.16	<0.0001	0.0063	0.0899
**Rotarod test**									
Time (s)	183.3 ±17.74	214.3 ± 12.06	208.7 ± 18.82	85 ± 8.11	99.17 ± 15.3	169.2 ± 21.71	<0.0001	0.0003	0.0496
**Pole test**									
Descent time (s)	4.40 ± 0.20	5.01 ± 0.25	5.79 ± 0.77	8.68 ± 1.23	10.32 ± 1.12	6.84 ± 0.40	<0.0001	0.0005	0.0386

Data represented mean ± SEM, $p < 0.05$ was set as the threshold for significance by one-way ANOVA followed by post hoc comparisons using Tukey's test for multiple groups' comparisons.

Supplemental Figure legend

Supplemental Figure. 1 OCN administration had no effect on motor function, dopaminergic neurons, gut microbiota and SCFAs. (A) Bar plots of performance in the behavioral tests, including the open field test, pole test, cylinder test and rotarod test. n = 10 per group. (B) (Upper panel) Representative immunostaining showing TH-positive neurons in the SN. (Lower panel) The average number of TH-positive neurons in the ST. n = 3 per group, 3 sections per mouse.(C) (Left panel) Representative immunostaining (upper) and western blotting (lower) showing TH-positive fibers and TH protein levels in the striatum. (Right panel) The quantitation of TH-positive fibers (upper) and TH protein levels (lower) in the striatum. Immunostaining: n = 3 per group, 3 sections per mouse; western blotting: n = 3 per group. (D) (Upper panel) The plots of alpha and beta diversity. n=10. (Lower panel) Bar plots of the RAs of f_*S24-7*, f_*Rikenellaceae*, f_*Lachnospiraceae*, f_*unclassified Clostridiales* at the family level. n=10. (E) Bar plots of the RAs of K01847, K00634, K00929 and K00074, n = 10 per group. (F) Bar plots of comparing the fecal levels of acetate, propionate and butyrate among groups. n=10.

Supplemental Figure. 2 Antibiotic treatment depleted the gut microbiota. (A) The concentration of stool-extracted DNA between control group and Abx group. n=4. (B) The comparison of cecal size between control group and Abx group. (C) The comparison of Stool cultures between control group and Abx group.

Supplemental Figure. 3 The comparison of alpha diversity of gut microbiota in 6-OHDA-induced PD mice. (Left panel) Bar plot of the Shannon index. (Right panel) Bar plot of the Simpson index. n-7-12, The data represent the mean ± SEM and was analyzed by one-way ANOVA followed by post hoc comparisons using Tukey’s test for multiple groups’ comparisons.

Supplemental Figure. 4 Cisplatin ablated the enteric neurons. (Left panel) The Representative immunostaining showing PGP9.5-positive neurons in the ileum between control group and cisplatin-treated group. (Right panel) The average number of PGP9.5-positive neurons in the ileum. n-3-5, The data represent the mean ± SEM

and was analyzed by one-way ANOVA followed by post hoc comparisons using Tukey's test for multiple groups' comparisons.

Supplemental Fig. 5 Correlation analysis between the altered KO and gut microbial taxa. Spearman correlation analysis of RAs of K01847 and that of p_*Bacteroidetes*, A; *f_S24-7*, B; f_*Rikenellaceae*, C; p_*Firmicutes*, D; f_*Lachnospiraceae*, E and f_*unclassified Clostridiales*, F. $p < 0.05$ was set as the threshold for significance.

Supplemental Fig. 1

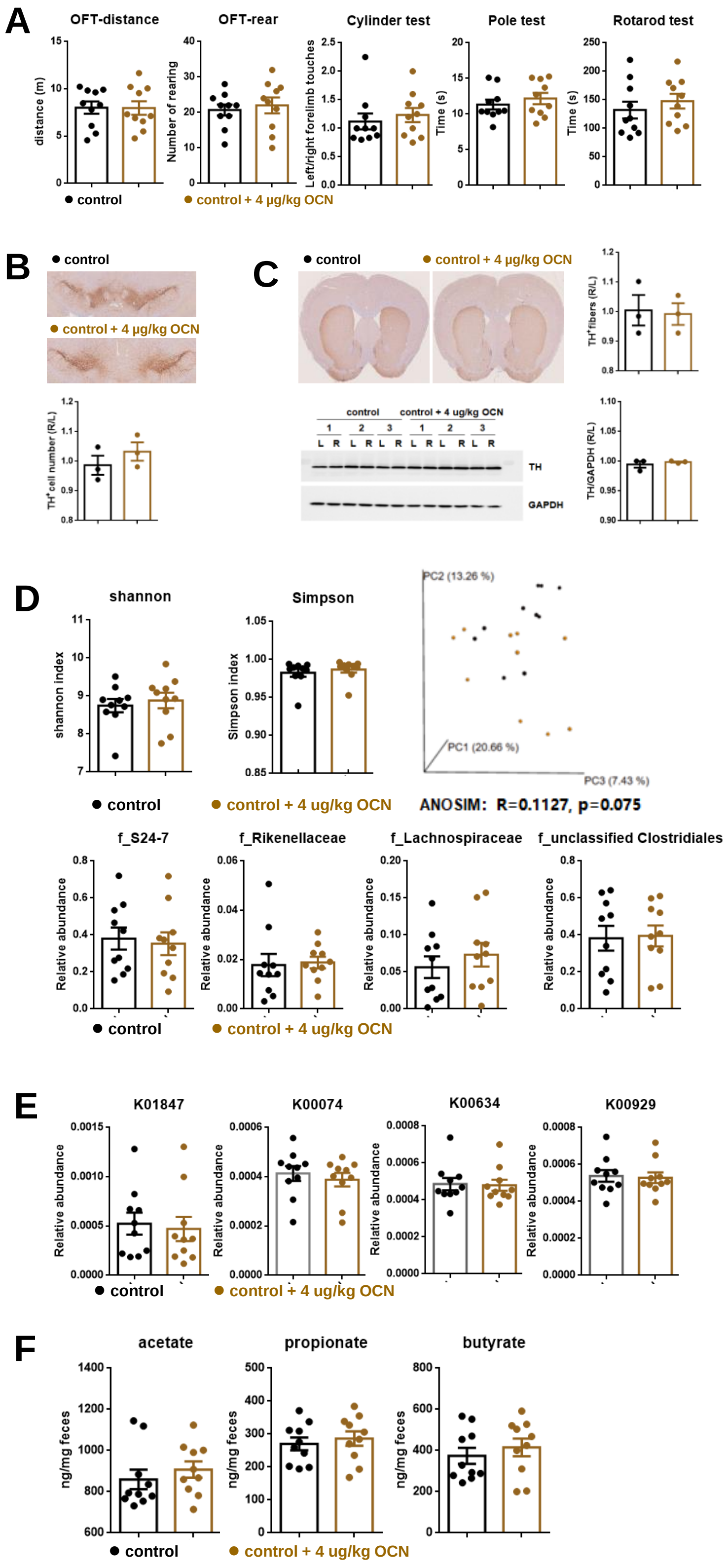

Supplemental Fig. 2

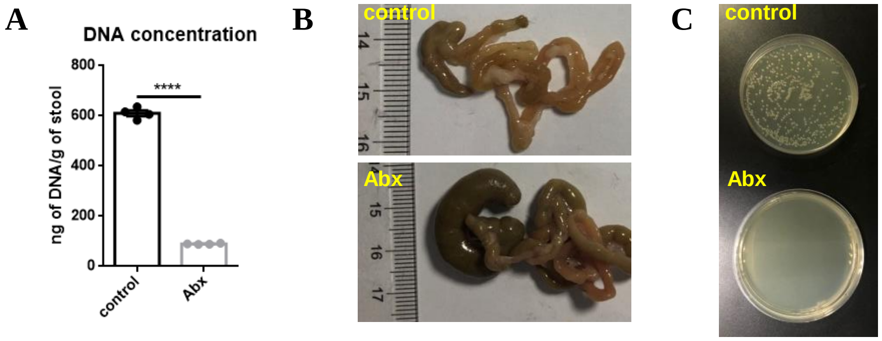

Supplemental Figure. 2 Antibiotic treatment depleted the gut microbiota. (A) The concentration of stool-extracted DNA between control group and Abx group. n=4. (B) The comparison of cecal size between control group and Abx group. (C) The comparison of Stool cultures between control group and Abx group.

Supplemental Fig. 3

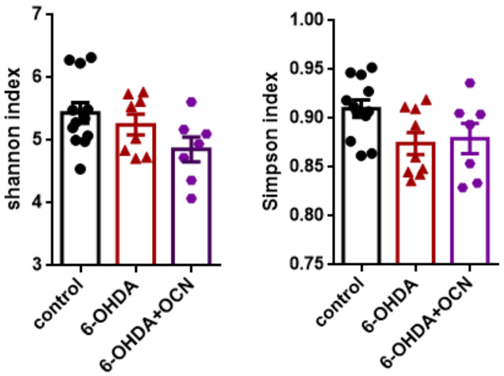

Supplemental Figure. 3 The comparison of alpha diversity of gut microbiota in 6-OHDA-induced PD mice. (Left panel) Bar plot of the Shannon index. (Right panel) Bar plot of the Simpson index. n-7-12, The data represent the mean ± SEM and was analyzed by one-way ANOVA followed by post hoc comparisons using Tukey’s test for multiple groups’ comparisons.

Supplemental Fig. 4

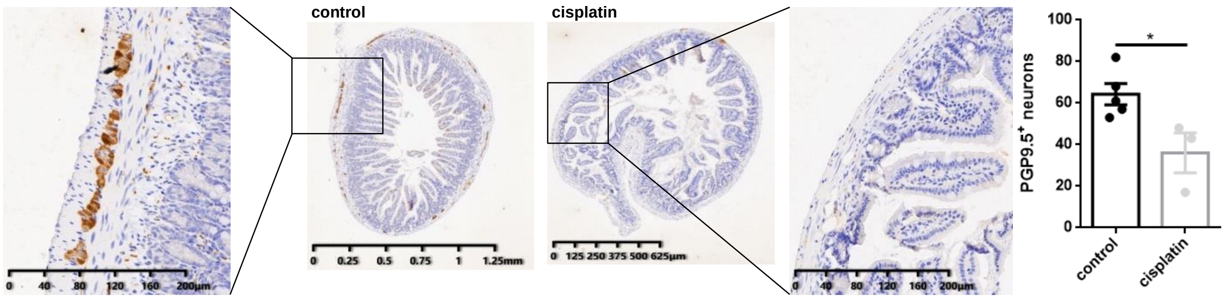

Supplemental Figure. 4 Cisplatin ablated the enteric neurons. (Left panel) The Representative immunostaining showing PGP9.5-positive neurons in the ileum between control group and cisplatin-treated group. (Right panel) The average number of PGP9.5-positive neurons in the ileum. n-3-5, The data represent the mean ± SEM and was analyzed by one-way ANOVA followed by post hoc comparisons using Tukey's test for multiple groups' comparisons.

Supplemental Fig. 5

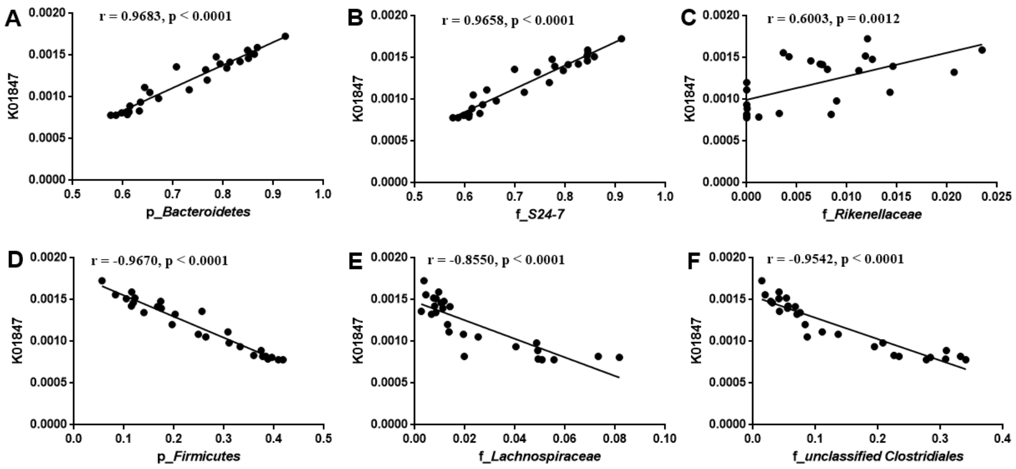

Supplement: Supplementary file 2 — Additional file 1: Supplemental Table S1. The comparison of the behavioral tests in 6-OHDA-induced PD mice after the administration of OCN. Supplemental Table S2. The comparison of the behavioral tests in 6-OHDA-induced PD mice treated with OCN after gut microbiota-depletion. Supplemental Table S3. The comparison of the behavioral tests after fecal microbiota transplantation. Supplemental Table S4. The overall OTU numbers and annotation levels. Supplemental Table S5. The comparison of taxonomy RAs of gut microbiota in 6-OHDA-induced mice after the administration of OCN. Supplemental Table S6. The comparison of RAs of KOs regulating SCFA metabolism in 6-OHDA-induced mice after the administration of OCN. Supplemental Table S7. The comparison of the behavioral tests in 6-OHDA-induced mice after the administration of propionate. Supplemental Table S8. The comparison of the behavioral tests in 6-OHDA-induced mice after the administration of FFAR3 agonist. Supplemental Figure 1. OCN administration had no effect on motor function, dopaminergic neurons, gut microbiota and SCFAs. Supplemental Figure 2. Antibiotic treatment depleted the gut microbiota. Supplemental Figure 3. The comparison of alpha diversity of gut microbiota in 6-OHDA-induced PD mice. Supplemental Figure 4. Cisplatin ablated the enteric neurons. Supplemental Figure 5. Correlation analysis between the altered KO and gut microbial taxa. [file 40168_2020_988_MOESM2_ESM.pdf]
